# Improving access to and effectiveness of mental health care for personality disorders: the guideline-informed treatment for personality disorders (GIT-PD) initiative in the Netherlands

**DOI:** 10.1186/s40479-020-00133-7

**Published:** 2020-08-10

**Authors:** Joost Hutsebaut, Ellen Willemsen, Nathan Bachrach, Rien Van

**Affiliations:** 1 Viersprong Institute for Studies on Personality Disorders, Halsteren, The Netherlands; 2 Centre of Expertise on Personality Disorders, Utrecht, The Netherlands; 3 Parnassia Psychomedical centre, The Hague, The Netherlands; 4 GGZ Oost Brabant, Boekel, The Netherlands; 5 Rino Zuid, Eindhoven, The Netherlands; 6 Tilburg University, Tilburg, The Netherlands; 7 Arkin Mental Health Centre, Amsterdam, The Netherlands

**Keywords:** Personality disorders, Generalist treatment, Implementation, Dissemination, Common factors

## Abstract

Evidence-based treatment for patients suffering from personality disorders (PDs) is only available to a limited extend in the Netherlands. Consequently, most patients receive non-manualized, unspecialized care.

This manuscript describes the background, rationale and design of the Guideline-Informed Treatment for Personality Disorders (GIT-PD) initiative. GIT-PD aims to provide a simple, principle-driven, ‘common-factors’ framework for the treatment of PDs. The GIT-PD framework integrates scientific knowledge, professional expertise and patient experience to design a good-enough practice, based on common factors. It offers a basic framework including general principles, a structured clinical pathway, a basic professional stance, interventions focused on common factors, and team and organizational strategies, based on common features of evidence-based treatments and generic competences of professionals.

The GIT-PD initiative has had a large impact on the organization of treatment for PDs in the Netherlands. For countries with an interest in improving their health care system for PDs, it could serve as a template that requires only limited resources.

## Background

Personality disorders (PDs) are a highly prevalent mental condition world-wide, associated with lifelong social and professional disability [1, 2], reduced life expectancy [3], and high societal and health care costs [4]. Following a range of studies showing effectiveness of psychotherapy for PD patients [5], there now is a rather optimistic view of the treatability of PDs. Several national guidelines recommend one of the evidence-based psychotherapy programs, such as Dialectical Behavior Therapy, Schema Therapy and Mentalization-Based Treatment [6–8]. However, implementation of these guidelines in clinical practice is cumbersome and most PD patients do not receive psychotherapy [9]. This is mainly due to capacity problems: delivering specialist psychotherapy is expensive and there is a lack of sufficiently trained professionals due to long training trajectories [10, 11]. Various studies also indicate that the sustainability of highly specialized evidence-based psychotherapy programs both for PD and for other complex disorders in the mental health services is often not guaranteed [12–14]. As a consequence, even in a country like the Netherlands, with relatively accessible mental health care services, only 23% of Borderline PD (BPD) patients received some sort of psychotherapy, not necessarily evidence-based, and usually in a lower dosage than recommended by the guidelines [10]. The large majority of PD patients therefore receives non-manualized “treatment as usual”, of which the content, duration, dosage and setting can vary widely. This limits the overall effectiveness of PD treatments, which may be reflected in the ongoing high consumption of different sorts of care by PD patients [15]. From a health economics point of view, improving access to psychotherapeutically informed care for PD patients may be a cost-effective strategy. The annual cost of PDs in Europe is estimated as in excess of € 25 billion [16] and several studies have demonstrated cost-effectiveness of psychotherapies for PDs [4, 17, 18].

In order to provide an accessible and feasible service for PDs, the Guideline-Informed Treatment for Personality Disorders (GIT-PD) project was started in the Netherlands. The project has realized a significant reform of health care services for PDs, providing an evidence-informed framework based on common features of specialized psychotherapies for treatment of PDs. Compared to the theory driven approach of specialist treatments, GIT-PD takes a rather pragmatic approach, focusing on common principles and stressing the organizational requirements for delivering good care for PD patients. In addition, GIT-PD requires less training and it upgrades existing services rather than that it completely reforms them, which may add to a simpler implementation. In this paper we present the general design and focus on organizational requirements and strategies for a successful implementation of this service-reform.

### Rationale of GIT-PD

Despite differences in theoretical background and approach, all specialist psychotherapy programs seem to be roughly equally effective [19]. According to Weinberg and colleagues, this may point to the importance of common features across the evidence-based approaches for PD rather than their specific features [20]. Based upon an inspection of the respective manuals, these authors identified five features that may explain the similar effects: clearly structured treatment framework, active stance of the therapist, focus on therapist-patient relationship, the use of interventions aimed at exploring the relationship between emotions, thoughts and behavior, and interventions to support motivation for change. These common features may facilitate the workings of common factors in the treatment of PDs, including a focus on improving the alliance, a cooperative stance, and effectively repairing ruptures [21]. Even more interestingly, assuring these generic features within a generalist treatment approach for PD turned out to be equally effective as specialist psychotherapies across various trials [19, 22–24].

Stressing the importance of basic generic principles above adherence to complex specialist psychotherapy models may have three additional advantages. Firstly, it could facilitate widespread dissemination of good-enough treatment, as training and supervision requirements may be reduced [25]. Secondly, some studies suggest that adherence to specialist programs is troublesome, negatively affecting outcome [26, 27]. Simplifying treatment principles may therefore improve ease of application and adherence. Finally, general principles may be applied more easily across different types of PD, while specialist theory-driven models are often focused more specifically on specific types, mostly Borderline PD.

These findings offer opportunities to provide better access to effective treatment for patients suffering from PDs. Instead of following the sometimes cumbersome and costly process of implementing evidence-based specialist programs, general quality of PD treatment may be more efficiently improved by enhancing the level of care in existing services in accordance with a common features approach [22]. The central issues then become how these common features can be organized within an integrative framework and what training is needed to assure their application. To address this issue, the Centre of Expertise on Personality Disorders (CEPD), a network organization for mental health institutes in the Netherlands, initiated the GIT-PD project in 2012. The aim was to make services more compliant to general principles of good care for PD patients and enhance uniformity across services in the Netherlands.

## Development of GIT-PD

The development of the GIT-PD framework started with a review of specialist and generalist PD treatment manuals [22, 24] and assumed mechanisms of change [28]. Expert meetings were organized as focus groups to identify relevant features in the treatment of PDs. Specific attention was given to suitability for PD patients with severe mental illness given the experienced shortcomings of specialist treatments for this subgroup [10]. In a focus group of 15–20 professionals from 9 mental health care institutions, relevant principles and ingredients for effective care were discussed, based upon the available expertise and experience of participants. These principles were manualized and matched with outcomes of the literature search on general principles of good enough care for PDs, including upcoming generalist approaches like Structured Clinical Management [22] and Good Psychiatric Management [24]. This manual was presented in several rounds to the focus group until sufficient detail and consensus were obtained. Initial drafts of this manual were also presented and discussed with a representative group of PD patients and family members until consensus was reached. Both patients and family members stressed the importance of engaging relatives in all stages of treatment, which became one of the core components of the GIT-PD approach. As the manual is designed to allow a certain flexibility, new adaptations that were deemed necessary given developments in the field, could be introduced following the same process. This design reflected the ‘nature’ of the GIT-PD project: GIT-PD was designed top-down (using literature review) as well as bottom-up (using discussion in focus groups) in order to optimize an ongoing exchange of ideas by experts, professionals, patients and family members.

### Theoretical and clinical starting points

Following the DSM-5 Alternative Model for PDs, GIT-PD assumes that all PD patients share impairments in personality functioning as reflected in Self and Interpersonal impairments [29]. Self-functioning refers to the experience of a unique identity and of a sense of self-direction. Interpersonal functioning refers to the capacity for empathy and intimacy. Specific features of GIT-PD are designed using this starting point. Firstly, PD patients’ proneness to become emotionally dysregulated as part of their identity-impairments, may lead to repeating crises, impacting upon their environment, including family and professionals. This could generate iatrogenic responses such as over-medication or rapid hospitalization. Moreover, PD patients’ impairment in self-direction may interfere with a stable commitment to change, motivation for treatment and ability to self-reflect to gain insight in their problems. Secondly, GIT-PD anticipates that empathy problems of PD patients will affect the therapist relationship and may interfere with patients’ ability to learn socially from others, given their distrust and related suspiciousness towards the intentions of caregivers [30]. Their inability to tolerate intense treatment relationships may lead to alliance ruptures. It can also lead to lead to unhelpful emotional reactions in professionals or teams. The combined effect of the impairments that constitute the core of PD patients’ problems, may interfere with the assumed working mechanisms of therapy, making any treatment less effective and, at times, even iatrogenic. GIT-PD focuses on securing principles that decrease the likelihood of these phenomena interfering with beneficial treatment processes.

### Core features of GIT-PD

Firstly, GIT-PD is a framework. Compared to other approaches it is not primarily theory driven and prescriptive in terms of setting, modalities or content of sessions. GIT-PD allows institutions to keep local context-based traditions, while still improving their services. It assumes that the core effectiveness lies in a set of principles, rather than in specific content based on a strict adherence to any specific theoretical concept of personality. This enables not only a personalized approach based on a process of shared decision making with the patient to select the most suitable interventions, but also optimizes response to actual needs of the patients and families during treatment. Secondly, GIT-PD assumes that basic therapist skills – part of any training of mental health care professionals – can suffice to provide good-enough treatment, when applied with a professional stance adjusted to the interpersonal hypersensitivity of most PD patients. Thirdly, GIT-PD integrates the findings that the organizational context may have a large impact on treatment effectiveness [26]. Therefore, principles are formulated also at the level of contextual and organizational conditions, team effectiveness, therapist attitude and treatment focus.

### Outline of the GIT-PD treatment principles

Table 1 summarizes the core features of the GIT-PD program. It contains the organizational requirements of the program, principles of the therapeutic process and shared common factors across treatments of PD patients, related to the core impairments of PD patients.

**Table 1:** General principles of GIT-PD

Level	Principle	Explanation
Organizational	Support	Sustained support of team by management board
	Structure	Predictability of program; e.g. schedule of appointments, availability of team, exchange of information, responsibilities of team members.
	Integration	Cooperation with other services, e.g. crisis and social services; within and between units
Treatment process	Phased & episodic	Structured in time (begin-middle-end) and possibly episodic to prevent relapses
	Goal-focused	cooperative agreement upon goals. Regular evaluation, leading to adaptations or (premature) termination.
Team	Complementary	Enabling integration of all perspectives in the team
	Reflective	Enabling reflection upon team culture and processes
	Supportive	Enabling mutual support to prevent iatrogenic actions
Common therapeutic factors	Self: Identity (autonomy-focused)	Strengthening autonomy; enhancing self-esteem; using strategies to identify, regulate and cope with (intense) emotions, including crisis
	Self: Self-direction (motivation-focused)	Monitoring and fostering motivation to engage and change in treatment; establishing strategies to improve self-reflection; Encouraging self-management of life stressors
	Interpersonal: Empathy (other/context-focused)	Monitoring, discussing and challenging patient’s experiences of others, including the therapist; focusing on understanding emotions and behavioral reactions of others
	Interpersonal: Intimacy (relationship-focused)	Enhancing trust in therapy; establishing an emotionally involved relationship; repairing ruptures; involving relatives and restoring openness to social learning
Basic stance of therapists	Curiously involved	Taking an investigative, curious, involved stance
	Supportive, Empathic	Taking an empathic, validating and supportive stance
	Transparent, authentic	Open to discuss actual events in the therapist relationship

The table could be read bottom-up: the heart of GIT-PD is a common basic professional stance, characterized by active involvement, careful exploration, validating feedback mirroring the therapist’s understanding of the patient’s affects and honest, authentic and transparent discussion. Using this stance, therapists using GIT-PD will continuously focus upon common areas of vulnerability of PD patients: strengthening their identity by helping them focus upon their needs, affects, opinions and values; strengthening their sense of agency by encouraging the pursuit of goals and values, and by helping them understand their behavior; enhancing understanding of others and relationships; establishing secure, mutual intimate relationships, within and outside treatment, facilitating renewed social learning.

Therapists are always embedded in a team and some principles are formulated to enhance team effectiveness. This includes appointing a team member who monitors the process of the team discussion and enables a reflective stance and the integration of all perspectives on treatment progress. GIT-PD encourages shared caseload and active cooperation between team members. As a rule, interventions that deviate from usual practice are discussed with peer team members as a way to prevent unthoughtful action caused by strong emotional reactions.

Furthermore, interventions by team members should be oriented towards specific goals, which should be evaluated regularly. The treatment process should be structured in time, reflected by the clinical pathway of GIT-PD (see below).

At top level, managerial commitment and consistency over time are essential for implementing good care for PDs. Therefore, organizational prerequisites are addressed as a general principle as well. They refer to the contextual conditions, adequate managerial support, integration with other services to provide consistency in approach, and clear structure of the program.

In our opinion, GIT-PD principles are not specific for PDs alone and may also be considered as generic principles of good care. However, we believe that the specific impairments of people with PDs, like their emotional and behavioral dysregulation and their interpersonal distrust, may jeopardize the adherence of professionals and teams to these principles. Moreover, we believe that if professionals fail to adhere to these generic principles, PD treatment may be affected more than treatment of other conditions would be. Ultimately, PD treatment may even become iatrogenic [31].

### Clinical pathways of GIT-PD

**Fig. 1:**
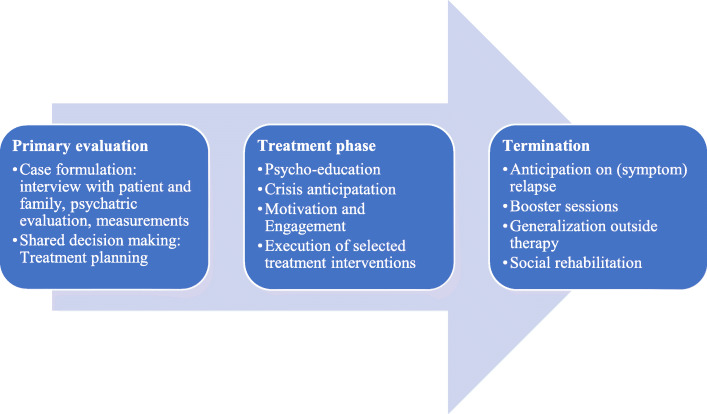
Clinical pathway of GIT-PD

GIT-PD stresses that treatment should be structured in time. This includes that treatment should be phased and specific targets for each phase should be identified. Figure 1 depicts the clinical pathway.

In the first phase careful assessment takes place, followed by discussion of core problems and determining the focus of treatment by therapist and patient. Treatment planning should be a process of shared decision making, tailoring treatment to the needs of the individual patient, thus promoting commitment to treatment and motivation for change.

The second phase (“treatment phase”) includes psychoeducation, crisis anticipation and execution of the selected treatment interventions. Ongoing attention to motivation and engagement is necessary. Review of treatment progress takes place on a regular basis. The third phase prepares for the termination of treatment. This phase includes anticipating possible relapsing symptoms, ‘booster’ sessions, usually in a lower frequency than in the treatment phase, and attention is being paid to recovery. Throughout all phases, the basic professional stance is maintained, focusing on collaboration and motivation.

### Supporting implementation by the Dutch Center of Expertise for personality disorders

Local mental health care units are responsible for the implementation of the GIT-PD framework. The Center of Expertise for Personality Disorders (CEPD) has provided a range of tools to support implementation and maintenance:

1. Online support and the GIT-PD website

The website contains information on GIT-PD, including procedures for frequently occurring clinical issues, like crisis management, drop-out, and boundary transgressions. It also includes around 50 demonstrational videos, that provide exemplary interventions for mental health care workers. An online toolbox contains descriptions of specific modules, psycho-educational leaflets, implementation plans and more. These tools have been supplied by participating mental health care centers. This is part of the earlier mentioned process of promoting exchange of ideas and materials between mental healthcare facilities.

2) Training course

The CEPD provides a 2-day ‘teach-the-teacher’ training, which discusses the GIT-PD principles and offers active training on basic interventions, including the basic stance, assessment and treatment review, managing emotions and crises, and repairing ruptures. In addition, the training focuses on educational and supervising skills, enabling participants to subsequently train their own teams. Training materials are free to use for institutions participating in the CEPD. The course thus enables professionals of participating institutes to optimize the GIT-PD approach in their own teams.

3) Template for managerial implementation

A template for managerial implementation is available, which is based upon the current state-of-the-art literature about implementation of change in care. This outlines several steps for successful implementation, like making a diagnostic organizational analysis, mapping possible risks, interventions to tackle risks, and systematic planning of process monitoring. This template is offered as a support tool, but implementation itself is done by the local units.

4) Quality maintenance

Quality maintenance is provided through audits of participating services, organized and facilitated by the CEPD. Mental health care units are clustered into small groups. Professionals of mental healthcare units visit each other every 2 years and assess quality of care by using the GIT-PD principles as criteria.

## Discussion

This paper discussed the GIT-PD initiative in the Netherlands. Essentially, GIT-PD strips down the complexities of specialist evidence-based treatment approaches to keep the most accessible and valuable basic principles of good-enough care for PD patients. The GIT-PD framework delivers practicable principles that can guide management of PD patients at the level of the therapist, the team and the organization. This framework is designed to provide a framework that is easy to implement and viable to adhere to over a longer time, helping to provide good-enough treatment for a broad group of patients with PDs. It was designed as an alternative for the prevailing *treatment as usual* given the often-insufficient capacity of specialist evidence-based programs. Additionally, as the GIT-PD principles are addressing the core impairments of PDs, it may provide an evidence-informed alternative for the treatment of the broad range of PDs. Ultimately, we believe both specialist and generalist approaches should play their role in the treatment of PDs, either in a stepped or matched care approach. Since the introduction of GIT-PD, there is tendency shift towards specialist psychotherapeutic centers in the Netherlands using GIT-PD as a basic treatment program for the majority of their patients, while reserving their specialist (MBT, DBT and SFT) programs for more complex patients who often have had an extended history of treatment failures.

### Key factors in the successful reform of PD services

The GIT-PD project was initiated in 2012, starting with 8 mental health care centers. Now, 8 years from the start, 20 large Dutch health care institutions participate, and they have restructured their PD treatment units according to the GIT-PD principles. This reform has made psychotherapeutically informed treatment available to a larger and broader range of PD patients across the country.

We believe several factors have contributed to this success. GIT-PD was partly designed ‘bottom-up’ and professionals of all participating institutions and patients have been closely involved, also in the implementation phase across the institutes. GIT-PD is not strongly theory driven nor is it prescriptive allowing institutions to follow local preferences and methods.

Training therapists in specialist psychotherapies is a costly and long-term investment that often runs into budgetary constraints. GIT-PD offers an easier and cheaper way to provide basic skills for the treatment of PD patients. Moreover, training programs for specialist psychotherapies are reserved for registered psychotherapists (psychologists/psychiatrists), while most professionals in the field are social workers or nurses. The upgrading of existing services for PD has highly supported professionals and gave them an increased sense of agency in their work with this difficult-to-treat patient group.

Finally, although specialist treatment programs in the Netherlands now tend to include more severely impaired PD patients with a history of previously failed treatments, still the most severe mentally ill PD patients are excluded from these programs, as they suffer from too many crises, disrupting social problems or because they lack basic psychological mindedness. Consequently, treatments for these severely ill patients often lack any structure and methodological coherence. Due to a flexible delivery of interventions, the team approach and contextual support, GIT-PD provides a better alternative for these patients than the existing services.

### Similar initiatives

Given the high societal costs associated with mental disorders, the improvement of access to effective psychological treatment is a major challenge for mental health care world-wide. In England, the ‘Improving Access to Psychological Therapies’ (IAPT) initiative by the National Health Service (NHS) aims to make evidence-based treatment for anxiety and depressive disorders available to a larger number of patients [32]. In Australia, the Project Air Strategy for Personality disorders is a partnership between the University of Wollongong and the NSW Ministry for Health, aiming to support better treatments for personality disorders [33]. What both projects have in common with GIT-PD are a dissemination strategy, training opportunities, and supervision of participating therapists. It should be noted however, that IAPT aims at improving treatment outcome for anxiety disorders and depression by promoting specific types of evidence-based treatment, linked to scientific evaluation from the start. Project Air and GIT-PD have both been developed to provide better evidence-informed care by encouraging cooperation among services, professionals, families and patients. The focus in both projects so far is on the organizational requirements that services need to deliver beneficial and sustainable treatment programs for patients with PD. However, unlike Project Air, the GIT-PD project was initiated by the local mental health institutions and is also fully financed by these services without additional funding.

### Limitations

The aim of the GIT-PD project is to improve treatment as usual by identifying common features of evidence-based treatment approaches and by implementing pragmatic principles related to good care for PDs. Although inter-institutional audits are conducted regularly to improve quality, data are not collected in a way that allows the monitoring of the impact on adherence and outcomes in clinical practice. Therefore, the GIT-PD project lacks scientific evaluation and evidence of the effectiveness in clinical practice is not yet available. As described, we believe the success of the GIT-PD lies in its flexibility and its personalized approach for patients and their relatives. However, this also generates a heterogeneity that may complicate systematic evaluation in commonly applied research designs. Nevertheless, we believe future studies could use a template GIT-PD program to study its effectiveness compared to specialist psychotherapy programs. Alternatively, aggregated data from routine outcome monitoring could be used if institutions are willing to harmonize data collection protocols.

## Conclusion

GIT-PD has become a widespread and well-known standard treatment approach in the PD field in the Netherlands. Almost all major mental health centers participate in the project on a voluntarily basis forming a network structure supported by the national CEPD. We hope the GIT-PD project can serve as an attainable and cost-effective template for other countries as well in order to improve the general quality of care for the large group of patients suffering from severe personality pathology.^1^

## Data Availability

NA

## Footnotes

1 People who may be interested in some of the (subtitled and translated) materials can contact the first (J.H.) or second (E.W.) author.
